# Biocontrol Agents and Natural Compounds against Mycotoxinogenic Fungi

**DOI:** 10.3390/toxins12060353

**Published:** 2020-05-28

**Authors:** Selma Pascale Snini, Florence Mathieu

**Affiliations:** Laboratoire de Génie Chimique, Université de Toulouse, CNRS, 31326 Toulouse, France; selma.snini@toulouse-inp.fr

Mycotoxins are toxic fungal secondary metabolites that contaminate food and feed. Mycotoxin contamination occurs as soon as environmental conditions are favorable for fungal growth and mycotoxin production, in the fields, during storage of raw materials and during industrial processes. To reduce mycotoxin contamination, several methods could then be adopted at these different stages. These methods can either reduce fungal growth or directly reduce the mycotoxin amount. For several years, the use of phytopharmaceutical products was favored to reduce fungal infection and thus mycotoxin contamination. However, they present numerous disadvantages such as detrimental effects in mammals, environmental contamination and subsequent strong impact on microbial biodiversity [1]. Moreover, the recurring application of fungicides could lead to the development of fungal resistance which would compromise disease control [2]. For several years, to reduce the use of such chemical products, alternative strategies based on biocontrol agents (BCAs) or natural products have been investigated. 

Among mycotoxins, aflatoxin B1 (AFB1), mainly produced by *Aspergillus flavus*, is the most potent naturally occurring carcinogen and causes human hepatocarcinoma. Currently, to reduce AFB1 contamination in the fields, the use of atoxigenic strains is the most commonly used biological control method. In their study, Savić et al. isolated a native atoxigenic *A. flavus* strain from maize grown in Serbia and used it to produce a biocontrol product. The efficiency of the biocontrol product was evaluated in maize Serbian fields over two years. The results demonstrated that the biocontrol treatment had a highly significant effect in reducing total aflatoxin contamination by 73% [3]. While *A. flavus* is a saprophytic fungus, cereal crops can also be infected by phytopathogens which produce mycotoxins. Among them, the genus *Fusarium* is the most prevalent and represents a significant risk. To date, in Europe, for *Fusarium* spp, only two BCAs are available. To fill this lack of BCAs against *Fusarium* spp, Pellan et al. selected three commercial BCAs with contrasting uses and microorganism types (*Trichoderma asperellum*, *Streptomyces griseoviridis*, *Pythium oligandrum*) and studied their effect on *Fusarium graminearum* and *Fusarium verticillioides* growth and mycotoxin production. They observed variable levels of mycotoxin production and growth reduction depending on the BCA or the culture conditions, suggesting contrasting biocontrol mechanisms [4]. 

In addition to BCAs, microbial culture supernatants or extracts can also be used to reduce mycotoxin contamination. Indeed, microorganisms can produce several kinds of metabolites with biological activities. In this context, Zeidan et al. explored the antifungal potential of a Qatari strain of *Burkholderia cepacia* (QBC03). Their results demonstrate that this strain exhibits antifungal activity against a wide range of fungi belonging to the *Aspergillus*, *Fusarium* and *Penicillium* genera. Moreover, the addition of the *B. cepacia* culture supernatant (2.5% to 15.5%) in the culture medium drastically reduces the fungal growth of *Penicillium verrucosum*, *Aspergillus carbonarius* and *Fusarium culmorum*. Further studies will be conducted to decipher the precise mechanism of action of the antifungal compounds secreted by this *B. cepacia* strain [5]. In the same way, Hanif et al. demonstrated that fengycin extracted from *Bacillus amyloliquefaciens FZB42* inhibits *F. graminearum* growth and mycotoxin production [6]. Similar to microbial metabolites, natural compounds can also affect fungal growth and mycotoxin production. They are extracted from plants and they can be used as aqueous extracts, organic extracts or essential oils. Wang et al. demonstrated that citral essential oil completely suppressed the mycelial growth of *Alternaria alternata* at the concentration of 222.5 µg/mL, which is the minimal inhibitory concentration (MIC). Moreover, the 1/2 MIC of this essential oil inhibits more than 97% of the mycotoxin amount. A comparative transcriptomic analysis of *A. alternata* treated or untreated revealed that citral affects transcription of genes involved in alternariol biosynthesis [7]. In the same way, Degola et al. investigated the biological activity of *Citrullus colocynthis* stem, leaf and root extracts on *A. flavus.* Among the tested tissues, leaf and root extracts showed the highest levels of AFB1 reduction (up to 80% reduction) [8].

BCAs can also be applied during industrial processes to limit fungal growth and mycotoxin contamination. As an example, in the brewing process, *Geotricum candidum*, a filamentous yeast is used to reduce *Fusarium* spp. growth and the T-2 toxin concentration. Kawtharani et al. demonstrated that *G. candidum* produces phenyllactic acid at the early stages of growth, which is responsible for the reduction of the T-2 toxin concentration through the reduction in *Fusarium* spp. growth [9]. 

The last scientific article included in this Special Issue is on the fringe of the other articles and deals with the use of fullerol nanoparticles (FNP) to modulate the secondary metabolite profile of the most relevant foodborne mycotoxigenic fungi belonging to the genera *Aspergillus*, *Fusarium*, *Alternaria* and *Penicillium*. This is a preliminary study to present the proof of concept for the use of FNP against mycotoxin contamination. Thus, Kovac et al. demonstrated that exposure to FNP leads to the reduction in concentrations of 35 secondary metabolites depending on the concentration of the applied FNP and the fungal genus [10]. 
